# Effects of Prostaglandin Analogue Iloprost Treatment on Distant Organ Damage Following Ovarian Ischemia/Reperfusion Injury in Rats: An Experimental Study

**DOI:** 10.7759/cureus.8695

**Published:** 2020-06-19

**Authors:** Öznur Uludağ, Mevlüt Doğukan, Mehmet Duran, Ebru Annac

**Affiliations:** 1 Anesthesiology and Reanimation, Adıyaman University Faculty of Medicine, Adıyaman, TUR; 2 Anesthesiology and Reanimation, Adıyaman University Faculty of Medicine, Adiyaman, TUR; 3 Anesthesiology and Reanimation, Adiyaman University Education and Research Hospital, Adiyaman, TUR; 4 Histology and Embryology, Adıyaman University Faculty of Medicine, Adiyaman, TUR

**Keywords:** rat, iloprost, ischemia / reperfusion model, distant organ, heart

## Abstract

Background

Ischemia/reperfusion (I/R) injury causes oxidative stress, which, in turn, may impair the oxidant/antioxidant balance in tissues and cause damage to the tissues. The local effects of I/R injury can be typically observed in the related organ while systemic effects can be observed predominantly in the heart, brain, lung, and kidney. In this study, we aimed to evaluate the effects of iloprost on heart tissues after an ovarian I/R injury in an experimental rat model.

Materials and methods

A total of 32 female Sprague Dawley rats were used for the experiment. The rats were divided into four groups with eight rats each: Group I, control group; Group II, ischemia group; Group III, I/R group; Group IV, I/R + iloprost group. Surgical intervention was performed in each group and after the procedures, heart tissues were obtained and examined histopathologically.

Results

No significant pathological finding was found in Group I and II while degeneration of muscle fibers and interstitial edema was observed in group III and dilation of the vessels was detected in Group IV. No fibrosis or inflammation was observed in any group.

Conclusion

Iloprost provided protection against I/R injury and thus may be an alternative treatment for I/R injury.

## Introduction

Ischemia, or insufficiency of perfusion, is defined as restricted blood supply to tissues, causing a shortage of oxygen and other metabolites necessary for cellular metabolism. Acute ischemia is a clinical problem with a high morbidity and mortality risk [1]. The severity of ischemic damage is proportional to the duration and amount of hypoperfusion and varies according to cell type, sensitivity, blood requirement, and metabolism. In ischemia, lack of oxygen causes a shift to anaerobic metabolism, leading to impaired removal of metabolic waste products. Moreover, the release of cellular energy stores and the accumulation of toxic metabolites result in cell death [2]. Blood flow in ischemic tissue should be restored for cell regeneration and the removal of toxic metabolites. However, reperfusion of ischemic tissue itself paradoxically causes tissue damage, leading to the onset of a series of events [3].

Ovarian torsion is the rotation of the ovary around its own ligaments and is typically treated by detorsion [4]. Following detorsion, ovarian tissues are irrigated with oxygenated blood. Ischemia stimulates the migration of polymorphonuclear leukocytes and platelets into the ischemic tissue, thereby increasing the formation of inflammatory cytokines and free oxygen radicals. In turn, free oxygen radicals cause mitochondria and peroxidation of lipids in cell membranes, ultimately leading to ischemia/reperfusion (I/R) injury in ischemic tissues through the activation of leukocytes and reactive oxygen metabolites [5].

Literature indicates that I/R injury can be more harmful than ischemic damage [5-6]. Moreover, I/R injury leads to both local and systemic effects, of which local effects are observed in the affected organ and systemic effects can be observed predominantly in the heart, brain, lung, and kidney [7]. The distant organ effects of I/R injury may lead to the development of septic shock and multiple organ dysfunction syndrome (MODS). Central to I/R injury are the free oxygen radicals resulting from the oxygenation of the tissue during reperfusion. Moreover, inflammatory response characterized by leukocyte activation plays the most important role in distant organ damage [8-9].

Prostacyclins are arachidonic acid metabolites used in the treatment of peripheral arterial diseases due to their vasodilatory and antiaggregant effects. Iloprost is a prostacyclin derivative that exerts its vasodilatory effects through prostaglandin pathways. Moreover, iloprost inhibits leukocyte activation and adhesion by reducing leukotriene, free oxygen radicals, and proteolytic enzyme secretion, thereby exerting its endothelial protective effect [10].

In the present study, we hypothesized that the heart tissues are affected by I/R injury occurring in any organ. Accordingly, we aimed to investigate the effects of iloprost on heart tissues through an experimental ovarian I/R model in rats.

## Materials and methods

Animals

A total of 32 female Sprague Dawley rats aged eight to 10 months, weighing 280-300 g were used in the study. All animals were exposed to a 12/12-hour light/dark cycle with the ambient temperature maintained at 22 ± 2 °C. All the experimental procedures were approved by the Adiyaman University Experimental Animal Research and Application Center Ethics Committee (Approval No: 13.12.2018/2018-30). All applicable international, national, and institutional guidelines for the care and use of animals were followed [11].

Experimental groups

The 32 rats were divided into four groups with eight rats each:

Group I (Control Group)

Midline laparotomy was performed alone, without any additional surgical intervention. Heart tissue specimens were collected for histopathological examination.

Group II (Ischemia Group)

A 2-cm midline incision was made in the lower abdomen. The adnexa containing the tube and ovarian tissues was rotated 360 degrees clockwise (torsion) to interrupt ovarian blood flow and then was fixed to the abdominal wall. The heart was surgically removed after three hours of ischemia.

Group III (I/R Group)

The rats underwent three hours of ischemia via ovarian torsion with subsequent three hours of detorsion (reperfusion period). After a total of six hours, the heart was surgically removed.

Group IV (I/R + Iloprost Infusion Group)

The rats underwent three hours of ischemia followed by an infusion of iloprost (10 µg/kg, IV; Ilomedin® (Schering, Berlin, Germany)) initiated 10 min before reperfusion of the catheter (intravenous (IV) cannula 24 g (yellow) 19 mm x 50 (Braun Vasofix; B. Braun Medical Ltd, Sheffield, UK) inserted into the jugular vein. Iloprost infusion was continued throughout the first 60 minutes of reperfusion, followed by reperfusion for two hours. After a total of six hours, the heart was surgically removed.

In each group, the rats were sacrificed after the completion of surgical procedures. Subsequently, heart tissues were fixed in 10% formaldehyde solution for histopathological examination.

Histopathological evaluation

Heart tissues were fixed in 10% formaldehyde solution and routine histological tissue monitoring was performed with alcohol, xylene, and Paraplast. Tissue samples were embedded in paraffin and then the specimens were sectioned into 5-mm-thick sections. The sections were stained with hematoxylin and eosin (H&E) and Masson’s trichrome (MT) staining for histopathological evaluation. Stained sections were examined using a microscope attached to the Carl Zeiss Axiocam (Germany) ERc5 digital camera.

## Results

**Figure 1:**
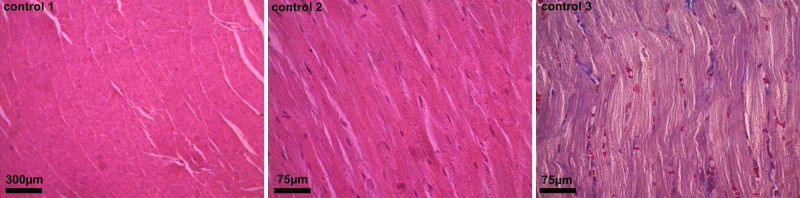
Light microscopy image of heart tissues of the control group (x10 - H&E staining, x40 - H&E staining, and x40 - Masson trichrome staining, respectively) Normal heart tissue image was observed H&E: hematoxylin and eosin

The examination of the control group revealed dense muscle fibers, no interstitial edema in the connective tissues between the fibers, no sign of dilation or hemorrhagic areas, and no pathological findings in muscle tissues (Figure 1).

**Figure 2:**
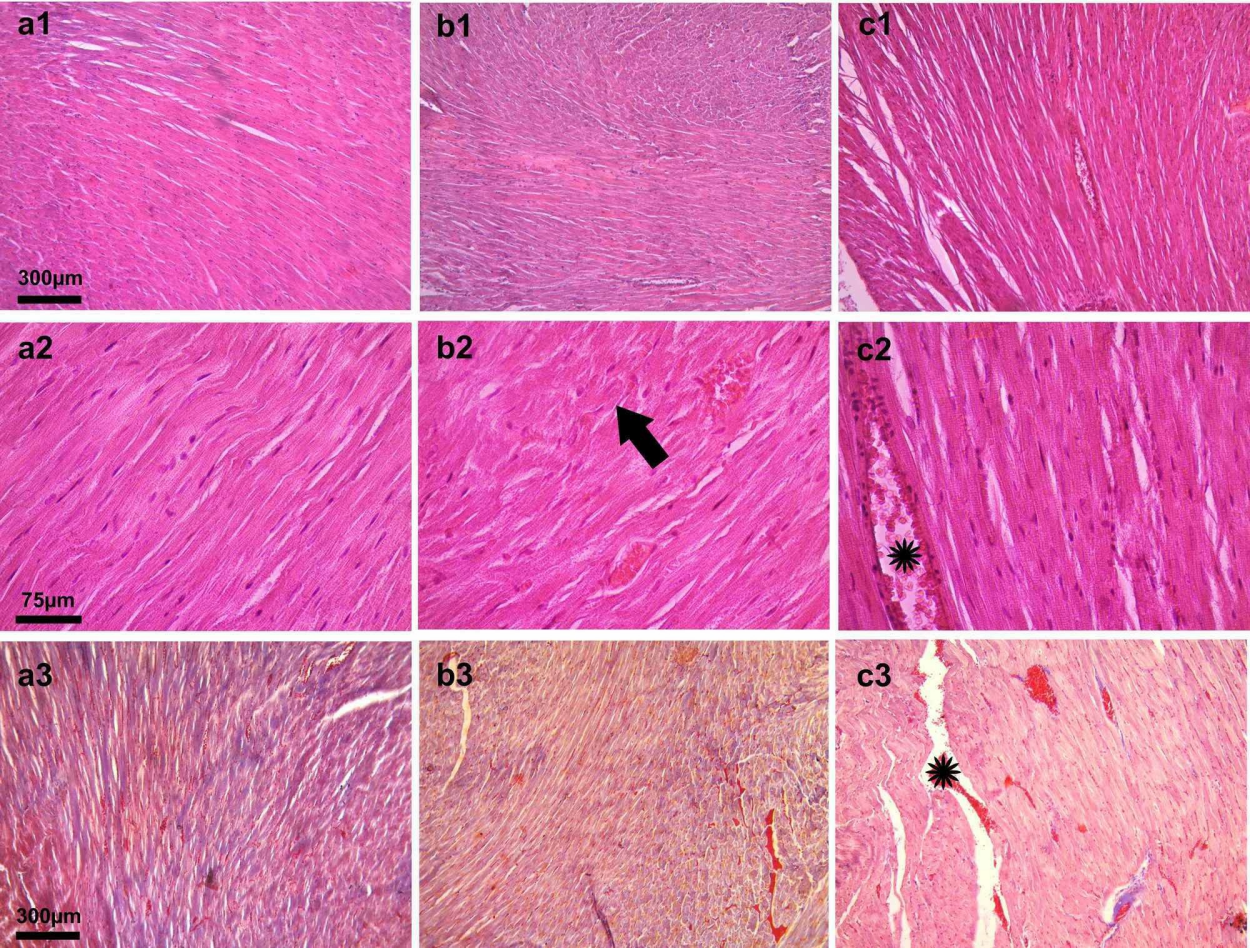
Light microscopic image of heart tissues of the ischemia group, I/R group, and I/R plus iloprost group, respectively a1, b1, and c1: Light microscopy image (x10, H&E staining) of the ischemia group, I/R group, and I/R plus iloprost group, respectively a2, b2, and c2: Light microscopy image (x40, H&E staining) of ischemia group, I/R group, and I/R plus iloprost group, respectively a3, b3, and c3: Light microscopy image (x10, Masson's trichrome staining) of ischemia group, I/R group, and I/R plus iloprost group, respectively Black arrow: deformed muscle fibers; star: dilated vein; H&E: hematoxylin and eosin

In the examination of Group II, the three layers of the heart (endocardium, myocardium, and epicardium) and normal-appearing heart tissue were observed (Figure 2; a1, a2, a3). In the evaluation of group III, myocardial damage, degeneration of muscle fibers, and interstitial edema were observed (Figure 2; b1, b2, b3). On the other hand, the evaluation of Group IV revealed the dilation of the vessels (Figure 2; c1, c2, c3).

**Figure 3:**
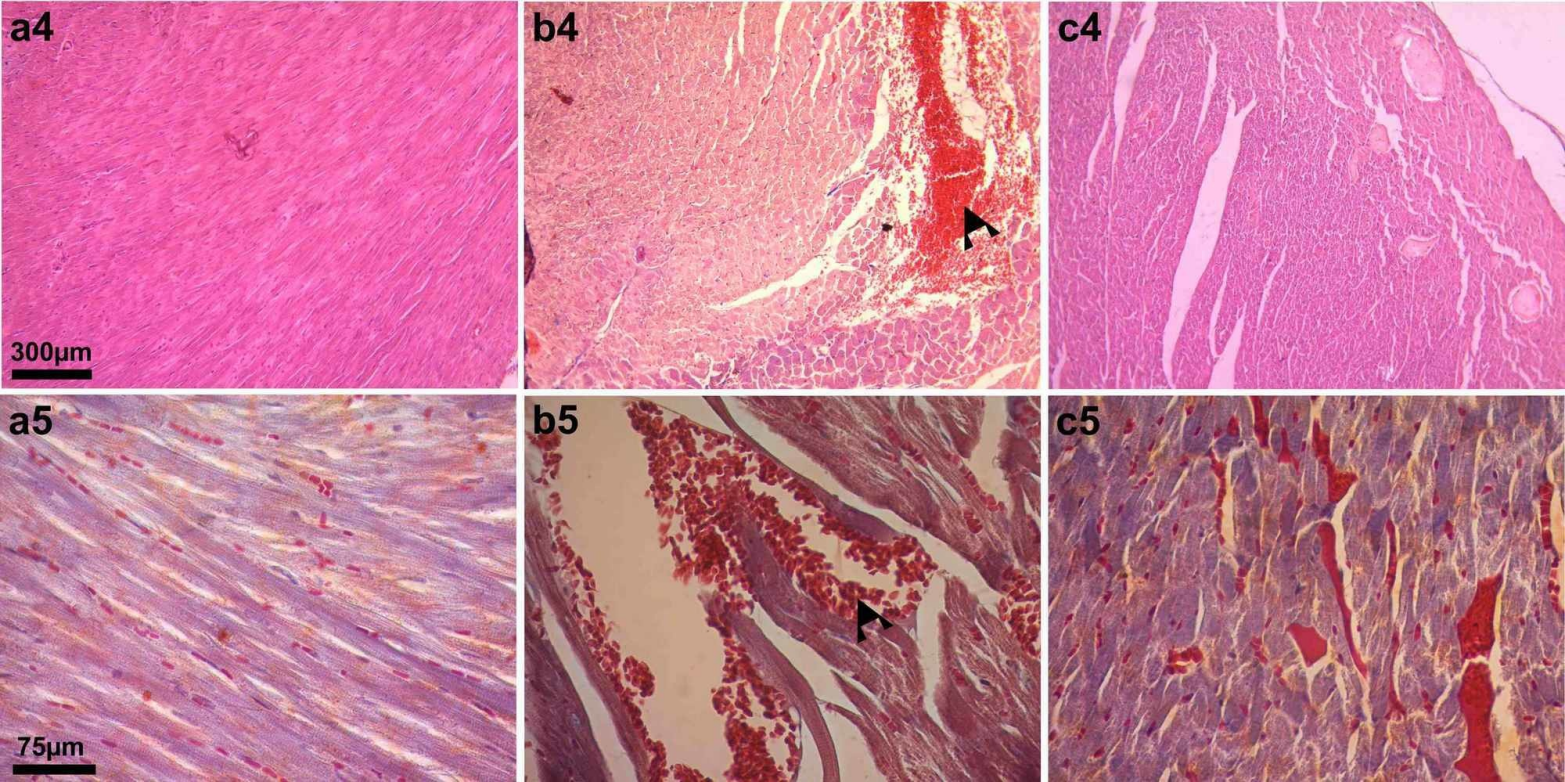
Light microscopic image of heart tissues belonging to the ischemia group, I/R group, and I/R plus iloprost group, respectively a4, b4, and c4: Light microscopy image (x10, H&E staining) of ischemia group, I/R group, and I/R plus iloprost group, respectively a5, b5, and c5, Light microscopy image (x40, Masson's trichrome staining) of ischemia group, I/R group, and I/R plus iloprost group, respectively Black arrowhead: hematological fields; H&E: hematoxylin and eosin: I/R: ischemia/reperfusion

The examination of the ischemia group (Group II) revealed normal heart tissue with dense muscle fibrils and a small number of connective tissues. The muscle fibrils had abundant sarcoplasm. Moreover, the nuclei of the cells were centrally located and were single and oval in shape. The connective tissues were remarkably thin and transparent (Figure 3; a4, a5).

In the I/R group (Group III), a degeneration of muscle fibers was observed when compared to the ischemia group. Additionally, interstitial edema and hemorrhagic areas were observed (Figure 3; b4, b5).

In the detailed examination of the I/R + iloprost group (Group IV), it was noted that the muscle fibril structure was generally similar to that of the ischemia group (Group II). In addition, vascular dilatations were observed (Figure 3; c4, c5). However, no fibrosis or inflammation was observed in any group.

## Discussion

A critical consequence of I/R is distant organ damage. Although the protective effect of iloprost on local organ damage has been shown experimentally, its effects on the distal organ in I/R injury have not been investigated sufficiently. The heart and lungs are the first organs to be affected by I/R injury [12]. In addition, I/R injury may lead to MODS or septic shock, which share similar clinical features, occur via inflammatory mediators, and potentially cause high mortality. Reperfusion also produces increased leukocyte traffic and vascular dysfunction in distant organs via complement system activation [13].

The morphological changes associated with prolonged ischemia followed by reperfusion include cellular swelling, membrane depolarization, loss of pinocytotic vesicles, endothelial basement membrane separation, and adherence of active leukocytes to the endothelial cell surface. Moreover, volume-controlled anion duct dysfunction is reported as the most important factor affecting volume swelling due to I/R injury [14].

The mainstay treatment of ischemia is increasing coronary blood flow, which in turn may lead to reperfusion damage. Therefore, once ischemia is detected, utmost care should be taken to prevent or minimize reperfusion injury. Currently, there are numerous clinical and pharmacological trials aiming to reduce reperfusion injury. Iloprost is a prostacyclin analog that can reduce I/R injury with its direct vasodilatory, cytoprotective, membrane stabilization, and antiaggregant and anti-inflammatory effects. Iloprost is also effective in local organ damage caused by I/R and has been shown to reduce renal I/R injury and to provide protection against lung injury caused by skeletal muscle I/R [15-17].

Some animal studies induced experimental ischemic damage without inducing reperfusion and reported that iloprost showed myocardial protective effects independently of the onset of ischemia. The studies also showed that iloprost preserved myocardial function in the myocardial I/R model and that the infarct area decreased more as the reperfusion period prolonged by iloprost application [18]. Iloprost has also been shown to be a potent vasodilator that particularly affects the dilation of arterioles and venules and to reduce the increased vascular permeability in the microcirculation [19]. Moreover, iloprost has been demonstrated to reduce oxidative muscle damage after I/R and this effect has been associated with decreased neutrophil activation, chemotaxis, and superoxide anion production [20].

In our study, no pathology was detected in the heart as a distant organ in the control and ischemia groups. In the remaining groups, however, degeneration of the muscle fibers and interstitial edema was observed in the I/R group and vasodilation was observed in the I/R + iloprost group. These findings implicate that iloprost decreases heart tissue injury secondary to ovarian I/R injury and that endothelium-dependent vasodilation is an indicator of I/R injury and iloprost treatment plays an important role in maintaining vasodilatory response.

Limitations of the study

The study only included a histological evaluation of I/R injury and did not include any biochemical and immunohistochemical evaluation, which might limit the generalizability of our results. However, we consider that future studies investigating the effects of iloprost on I/R injury in different tissues may benefit from the findings of our study.

## Conclusions

In the current study, iloprost was found to be histopathologically effective in I/R-induced distant organ damage in rats and may be an alternative treatment for I/R injury. Further clinical studies are needed to substantiate our findings.
